# The Spectrum of Renal Allograft Failure

**DOI:** 10.1371/journal.pone.0162278

**Published:** 2016-09-20

**Authors:** Sourabh Chand, David Atkinson, Clare Collins, David Briggs, Simon Ball, Adnan Sharif, Kassiani Skordilis, Bindu Vydianath, Desley Neil, Richard Borrows

**Affiliations:** 1 Department of Nephrology and Kidney Transplantation, Queen Elizabeth Hospital, Birmingham, United Kingdom; 2 Centre for Translational Inflammation Research, University of Birmingham, Birmingham, United Kingdom; 3 Renal Department, Royal Shrewsbury Hospital, Shropshire, United Kingdom; 4 Histocompatibility and Immunogenetics Laboratory, NHSBT Birmingham Centre, Vincent Drive, Edgbaston, Birmingham, United Kingdom; 5 Department of Renal Histopathology, Queen Elizabeth Hospital, Birmingham, United Kingdom; Johns Hopkins School of Medicine, UNITED STATES

## Abstract

**Background:**

Causes of “true” late kidney allograft failure remain unclear as study selection bias and limited follow-up risk incomplete representation of the spectrum.

**Methods:**

We evaluated all unselected graft failures from 2008–2014 (n = 171; 0–36 years post-transplantation) by contemporary classification of indication biopsies “proximate” to failure, DSA assessment, clinical and biochemical data.

**Results:**

The spectrum of graft failure changed markedly depending on the timing of allograft failure. Failures within the first year were most commonly attributed to technical failure, acute rejection (with T-cell mediated rejection [TCMR] dominating antibody-mediated rejection [ABMR]). Failures beyond a year were increasingly dominated by ABMR and ‘interstitial fibrosis with tubular atrophy’ without rejection, infection or recurrent disease (“IFTA”). Cases of IFTA associated with inflammation in non-scarred areas (compared with no inflammation or inflammation solely within scarred regions) were more commonly associated with episodes of prior rejection, late rejection and nonadherence, pointing to an alloimmune aetiology. Nonadherence and late rejection were common in ABMR and TCMR, particularly Acute Active ABMR. Acute Active ABMR and nonadherence were associated with younger age, faster functional decline, and less hyalinosis on biopsy. Chronic and Chronic Active ABMR were more commonly associated with Class II DSA. C1q-binding DSA, detected in 33% of ABMR episodes, were associated with shorter time to graft failure. Most non-biopsied patients were DSA-negative (16/21; 76.1%). Finally, twelve losses to recurrent disease were seen (16%).

**Conclusion:**

This data from an unselected population identifies IFTA alongside ABMR as a very important cause of true late graft failure, with nonadherence-associated TCMR as a phenomenon in some patients. It highlights clinical and immunological characteristics of ABMR subgroups, and should inform clinical practice and individualised patient care.

## Introduction

“Late” allograft failure represents a major hurdle in kidney transplantation [1,2]. Refinements in detection and identification of donor specific antibodies (DSA), and in histological interpretation, highlight the importance of antibody mediated rejection (ABMR) in this process [3–5]. Detailed and informative studies suggest ABMR as the major cause of late graft failure, with a limited role for T cell mediated rejection (TCMR) or progressive scarring in the absence of an inflammatory process (interstitial fibrosis/tubular atrophy;IFTA) [3–6].

But the question arises as to whether this is truly representative. Specifically, landmark studies from the Edmonton [3] and DeKAF study [5] groups showed the deleterious effect of antibody-associated inflammation within biopsies from “troubled transplants”. Yet these patients often experienced dramatic deterioration in function after years of stability, with role of non-adherence subsequently highlighted in expanded cohorts [4]. Whilst certainly representing one pathway to graft failure, broader relevance for patients without such trajectories of (dys)function, who may not necessarily be non-adherent, who may not undergo biopsy, and so may be under-represented in such studies, is more questionable.

The Mayo Clinic group [6] also identified the importance of ABMR, although this was based on histology review as DSA data was unavailable. A key strength of this study represented investigation of an incident cohort, yet this necessarily placed limits on follow-up duration, with approximately half of studied failures occurring within 4 years post-transplantation, and all within 10 years. The spectrum of causes of “true” late graft failure beyond this time remains incompletely defined.

In contrast, the Leuven group [7] suggested that for failures specifically beyond 5 years post-transplantation, IFTA *in the absence of prior or current specific diagnosis* was more common, with 12/39 (31%) indication biopsies showing this lesion. The authors acknowledged that most (76.1%) grafts failing beyond 5 years did not undergo biopsy within 2 years of failure, and electron microscopy and DSA evaluation were unavailable. Nevertheless, other studies of non-selected graft failures beyond 3 months post-transplantation showed DSA at the time of graft failure in only 9/69 (13%) patients [8].

In light of this conflicting information we adopted an alternative approach, not dissimilar to that of a previous study reporting non-immunological risk factors as drivers to late graft failures [9]. We used a “period analysis” to identify in detail the causes of all unselected graft failures, including comprehensive histological (including electron microscopy) and DSA evaluation (including C1q-binding DSA). We demonstrate the spectrum of causes of allograft failure from the post-operative period to many decades post-transplantation. The study confirms the importance of ABMR and refines clinico-pathological correlations in light of contemporary histological classification [10]. But we also demonstrate the importance of progressive scarring (IFTA) in the absence of other specific inflammatory diseases in unselected true late graft failures.

## Materials and Methods

### Approach and Rationale

We evaluated the clinical, histological and immunological data in all non-selected transplant failures occurring between 2008 and 2014 in patients at a single transplant centre. This represents an era during which liberal HLA antibody testing was undertaken using solid-phase screening and identification. In addition, we incorporated indication transplant biopsies more liberally in the program, often performed many years from transplantation when graft dysfunction may previously have been dismissed as inevitable and non-modifiable. Biopsies were undertaken at the discretion of the treating clinical team, in general based on unexplained renal dysfunction (~10% rise in creatinine), and/or worsening proteinuria.

The study was conducted according to the Declaration of Helsinki and Istanbul and following permission and approval from the local research and audit office of University Hospital Birmingham. In regard to the center’s clinical practice, none of the transplant donors were from a vulnerable population and all donors or next of kin provided written informed consent that was freely given.

### Histological, Immunological and Clinical Data

Pathologists with specific expertise in renal pathology reported all biopsies. Each biopsy qualifier was graded according to the Banff classification and electronically stored. For this study, these records were reviewed in the context of the recently updated Banff ABMR classification [10], the available DSA data, and the clinical scenario. Histological features of ABMR (microvascular inflammation; peritubular/glomerular basement membrane changes on electron microscopy; C4d deposition) were specifically recorded; polyclonal anti-C4d antibody staining (Biomedica) was used on formalin-fixed paraffin-embedded sections with low pH antigen retrieval; conventional staining (immunoperoxidase) was used to evaluate (recurrent or de novo) glomerulonephritis; glutaraldehyde fixation was used for electron microscopy sample processing.

Samples for circulating DSA were always tested at the time of biopsy and graft failure (return to dialysis or retransplantation), and at other times according to clinician approach. For patients returning to dialysis, testing was undertaken prior to immunosuppression weaning; for patients undergoing pre-emptive retransplantation, testing was undertaken prior to retransplantation. Solid-phase assays (Luminex) screened for and then subsequently identified specificities of circulating HLA antibodies (One Lambda). For patients demonstrating HLA antibodies, the presence of C1q-binding antibodies was evaluated using single flow bead assays according to manufacturers’ instructions (C1qScreen; One Lambda). Samples stored prior to transplantation were retrospectively tested to evaluate whether DSA was pre-existing or de novo post-transplantation.

Information regarding episodes of rejection, non-adherence, and measurements of estimated glomerular filtration rate (MDRD eGFR) and proteinuria (early morning “spot” urine albumin:creatinine ratio [ACR]) were obtained from clinical records and laboratory databases.

### Transplant Failure: timing and aetiology

Transplant failures were identified from the prospectively maintained departmental database, cross checked with data supplied by the National Registry (National Health Service Blood and Transplant; NHSBT). Only death-censored graft failures were evaluated. One hundred and seventy-one graft failure were identified. Twenty additional patients were identified by NHSBT whose grafts failure during this period but had left the region. Their demographics were similar to the 171 who underwent follow-up locally (data not shown) and were not studied further.

For perspective, during this period, the department followed a prevalent population of between 1200 and 1400 patients; between 2008 and 2014 the department undertook 978 transplant procedures; during this period a further 149 patients died with ongoing graft function. The 171 studies failures were then grouped depending on time to failure: within 1 month; 1 month to 1 year; 1–5 years; 5–10 years; >10 years.

The causes of graft failures were classified following a similar (but not identical) strategy to that of El-Zoghby [6]:

‘primary non-function’: usually resulting from renal transplant arterial or venous thrombosis. In a minority of cases (see “results”) kidney function insufficient to avoid dialysis, but in the absence of a defined “surgical”/”technical” cause was evident, probably representing severe preservation injury.

‘ABMR’: as defined in the recently updated Banff classification [10], and comprising the triad of circulating DSA, tissue injury, and evidence of antibody/endothelium interaction (based either on the magnitude of microcirculatory inflammation or C4d deposition; gene expression studies were not undertaken).

‘TCMR’: tubulo-interstitial immune infiltrates, with or without involvement of large vessels; microvascular inflammation (glomerulitis; peritubular capillaritis) in the absence of a detectable anti-HLA antibody was included in this category on the basis that these lesions are recognised previously as a nonspecific component of rejection [11,12]. It should be acknowledged though that these lesions may also represent ABMR as a result of non-HLA antibodies which were not evaluated in the current study. Therefore whilst it can be cogently argued that these lesions represent “possible ABMR”, aetiological certainty is impossible and so we have kept the label “TCMR” whilst highlighting the possibility of ABMR in some patients (a small minority) in the relevant section of “results”.

‘recurrent disease’: histological evidence of the same disease process that led to failure of the patient’s native kidneys

‘*de novo*’ glomerulonephritis was diagnosed in the context of a known primary nephropathy different to the disease seen in the transplant kidney

‘PVIN’: ‘polyoma virus interstitial nephropathy’; evidenced by characteristic viral inclusions on biopsy, positive staining for SV40 antigen, accompanied by circulating DNAemia. Graft failures from acute rejection following immunosuppression minimisation as treatment for PVIN were classified as PVIN, rather than rejection.

‘surgical’: resulting from vascular or ureteric complications beyond the immediate post-operative period.

‘medical’: transplant failure following an episode of acute kidney injury in the context of intercurrent severe illness without other defined renal aetiology.

‘IFTA’: interstitial fibrosis and tubular atrophy as the only abnormal histological finding, in the absence of other inflammatory or infective lesions described above.

### Timing of Transplant Biopsies

We primarily considered the biopsy taken most proximate to graft failure as most informative, or the first in a series of biopsies taken in quick succession (usually in the context of acute rejection). All biopsies were indication biopsies, and therefore should capture the prevailing (immune-) pathological scenario. Importantly, we set the following time limits in regard to using biopsies as “diagnostic”:

Within 2 months of graft failure for failures within 1 year

Within 6 months of graft failure for failures within 5 years

Within 12 months of graft failure for failures within 10 years

Within 24 months of graft failure for failures beyond 10 years

Although biopsies taken outside these timeframes were not considered diagnostic of graft failure, their findings are described where relevant and contributory. We acknowledge that not all patients underwent biopsy; nevertheless their clinical characteristics and DSA results may offer some insights and they are accordingly described in “results”.

### Statistical analysis

Data are presented as mean ± standard deviation unless stated otherwise. Continuously distributed data were compared using ANOVA and Students’ t-test, and categorical data by means of chi-squared testing. A type 1 error rate less than 5% (p≤0.05) was considered statistically significant.

## Results

### The spectrum of Graft Failure

**Fig 1:**
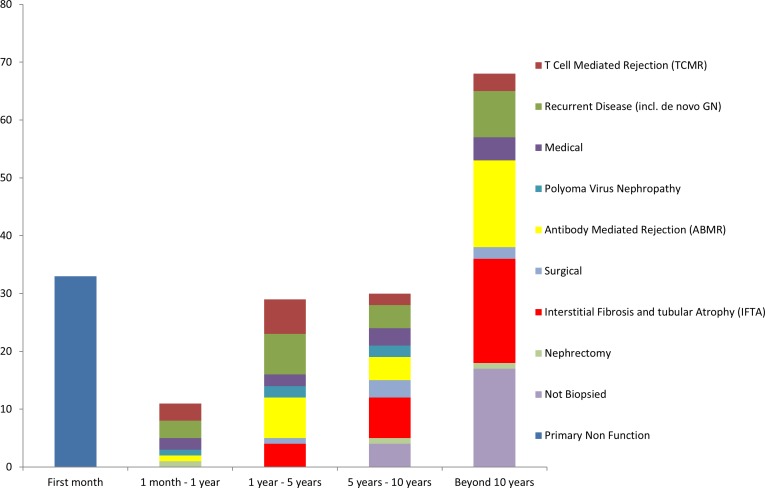
Causes of graft failure by period following transplantation.

Demographics of the 171 studied patients are shown in Table 1, and their causes of graft failure in Fig 1.

**Table 1:** Demographics of 171 studies patients experiencing graft failure.

Characteristic	
Age of Transplant (years)	38 ± 15
Sex	Male: 105; Female: 66
Ethnicity	White: 132; South Asian: 29; Black: 9; Chinese: 1
HLA Mismatch	2.6 ± 1.4 Ag
PRA at transplant (%)	Median: 0 (IQR 1–5)
Repeat Transplant	43
Donor Age (years)	45 ± 14
Live Donor	41
Donor following Cardiac Death	2
*Cause of Renal Faliure*	
Glomerular	49
Cystic	34
Diabetes	25
Hypertension	32
Other	21
*Date of Transplant*	
1990 and before	21
1991–1995	17
1996–2000	31
2001–2005	33
2006–2010	49
Since 2011	20
*Immunosuppression at transplantation*	
Prednisolone	160
Azathioprine	92
Mycophenolate	79
Ciclosporin	90
Anti CD25 MAb	94
*Time to Graft Failure*	
Average (days)	3103 ± 2927
Median (days)	2343 (IQR 127–5032)
Range (days)	0–13743 (37.6 years)

Early failures (within 1 month post-transplantation) were exclusively due to primary non function (n = 33), either vascular thrombosis (n = 28) or lack of function without identifiable cause and presumed either due to suboptimal “donor quality” or “preservation injury” (n = 5 with early biopsies showing acute tubular injury without inflammation or detectable DSA).

Subsequent graft failures were far more heterogeneous and included ABMR (n = 27), IFTA (n = 29), recurrent disease (n = 22), TCMR (n = 14), and PVIN (n = 5). Of the 22 cases of recurrent disease, 2 cases (both IgA nephropathy) were in the context of an unknown primary diagnosis. These probably represented recurrent disease, although *de novo* glomerulopathy could not be excluded.

‘Proximate’ biopsy data (timing defined in “methods”) was available in all 97 cases. The mean time between biopsy and graft failure was 4±2 weeks for failures within 1 year, 4±3 months for failures within 5 years, 7±4 months for failures within 10 years, and 14±5 months for failures beyond 10 years.

Medical complications (n = 11, usually severe sepsis), surgical complications beyond the immediate post-operative period (n = 6), and transplant nephrectomy for either PTLD or carcinoma (n = 3) were also contributors to graft failure; these grafts did not undergo biopsy.

Twenty-one grafts failed without undergoing “proximate” biopsy, representing 12.3% of all graft failures (21/171), and 17.7% of graft failures beyond a month, after excluding medical and surgical causes, and nephrectomies [21/(97+21)].

#### Antibody Mediated Rejection (ABMR)

Twenty-seven grafts failed to ABMR. DSA was present at the time of biopsy, and persisted to the time of graft failure; all DSA developed *de novo* post transplantation (exact timing unknown). Subclassification of graft failures was as follows: Acute Active ABMR (n = 9); Chronic Active ABMR (n = 15); Chronic ABMR (n = 3). All except 4 patients displayed microvascular damage (glomerulitis, peritubular capillaritis [PTCitis], transplant glomerulopathy or peritubular capillary basement membrane multilamellation [PTCBMML]) as histological qualifiers; 3 patients with Chronic Active ABMR and 1 patient with chronic ABMR displayed *macro*vascular changes (vasculitis and chronic intimal thickening respectively) without microvascular damage or inflammation.

Biopsies showing Acute Active ABMR displayed more glomerulitis and PTCitis than biopsies displaying Chronic Active ABMR (Table 2). All biopsies with Chronic Active ABMR displayed C4d staining; C4d staining was absent in 3/9 (33%) patients with Acute Active ABMR–these patients met criteria for Acute Active ABMR based on microcirculation inflammation in the absence of C4d staining. Biopsies displaying Chronic ABMR displayed numerically (but not statistically) more chronic glomerulopathy and PTCBMML.

**Table 2:** Characteristics of patients displaying ABMR on indication biopsy.

Characteristic	ABMR—Acute Active (n = 9)	ABMR—Chronic Active (n = 15)	ABMR—Chronic (n = 3)		p value	
Time to Graft Failure (days)	1936 ± 1857	5025 ± 2927	6446 ± 2536		0.01	
Age at transplant (years)	26 ± 5	27 ± 12	24 ± 5		NS	
Age at graft transplant (years)	31 ± 7	41 ± 11	41 ± 6		0.03	
eGFR 6 months prior to graft failure (ml/min)	42.6 ± 19.9	21.0 ± 7.4	16.0 ± 1.4		<0.001	
UACR at graft failure (mg/mmol)	37 ± 48	289 ± 193	72 ± 31		0.001	
Previous rejection	0	5 (33%)	0		0.04	
Non-adherence	6 (66%)	5 (33%)	0		0.002	
Class I DSA	4 (44%)	6 (40%)	1 (33%)		NS	
Class II DSA	1 (11%)	7 (47%)	2 (66%)		0.04	
Class I + II DSA	4 (44%)	2 (13%)	0		0.05	
C1q-binding DSA	4 (44%)	4 (27%)	1 (33%)		NS	
g-score	1.49 ± 0.82	0.47 ± 0.96	0		0.05	
ptc-score	1.56 ± 0.68	0.67 ± 0.94	0		0.03	
C4d	6 (66%)	15 (100%)	0		NS*	
cg-score	0	1.33 ± 0.88	2.33 ± 0.47		NS**	
PTCBMML	0	8 (53%)	3 (100%)		NS**	
ah- score	0.44 ± 0.68	2.30 ± 1.12	1.90 ± 0.94		0.001	
ci-score	1.66 ± 0.94	2.53 ± 0.61	2.30 ± 0.47		0.01	

*Comparison made excluding “Chronic ABMR” which by definition lacks C4d deposition (or would have otherwise been classified as “Chronic Active ABMR”);

**Comparison made excluding “Acute Active ABMR” which by definition lacks chronic histological qualifiers (cg or PTCBMML) or would have otherwise been classified as “Chronic Active A.

The characteristics of patients and DSA are also shown in Table 2. Time to graft failure was shorter, and age at graft failure younger, in patients with Acute Active ABMR compared with the other 2 histological groups. Faster decline in graft function (using eGFR 6 months prior to failure as a proxy) was also evident in the Acute Active ABMR group (excluding a single patient whose graft failed before 6 months post-transplantation). Non-adherence was noted in 66% of patients with Acute Active ABMR, compared with 33% with Chronic Active ABMR, and none with Chronic ABMR (p = 0.002).

Finally, ah-scores were lower in patients with Acute Active ABMR (Table 2); six displayed ah-scores of zero. Notable differences in ah-score were also seen between adherent and non-adherent patients with ABMR: 2.00±1.02 versus 0.25±0.66 (p<0.001).

#### DSA Class and C1q-binding DSA in ABMR

Class II DSA was more common in patients with Chronic Active or Chronic ABMR, compared with Acute Active ABMR (Table 2). No difference between groups for Class I DSA was evident.

Nine patients (33% of patients with ABMR) displayed C1q-binding DSA (class I n = 1; Class II n = 4;Both n = 4), with no difference across histological subgroups. Mean time to graft failure was shorter in patients with C1q-binding DSA (2454 versus 4759 days; p = 0.05).

#### Interstitial Fibrosis and Tubular Atrophy (IFTA)

Twenty-nine grafts failed with only IFTA on indication biopsy proximate to failure. None showed microcirculatory inflammation, vasculitis or C4d deposition; DSA was absent in all at the time of biopsy and at graft failure. Banff ci and ct scores were universally ≥2.

By definition, no cases displayed interstitial infiltrates meeting acute rejection criteria (including borderline change). However, milder interstitial inflammation was seen in some biopsies, as was inflammation in scarred areas. We therefore segregated IFTA cases into 3 groups (Table 3): IFTA without inflammation (n = 15); IFTA with inflammation confined to areas of scarring (n = 6); IFTA with inflammation outside scarred areas (n = 8).

**Table 3:** Characteristics of patients displaying IFTA on indication biopsy.

Characteristic	IFTA without inflammation (n = 15)	IFTA with inflammation in scarred areas (n = 6)	IFTA with inflammation in non-scarred areas (n = 8)	p value
Time to Graft Failure (days)	6101 ± 2147	4525 ± 2147	3325 ± 1867	0.002
Age at transplant (years)	31 ± 10	47 ± 11	28 ± 7	0.001
Age at graft transplant (years)	48 ± 10	61 ± 11	38 ± 9	<0.001
eGFR 6 months prior to graft failure (ml/min)	13.5 ± 1.5	15.8 ± 2.9	20.5 ± 5.5	<0.001
UACR at graft failure (mg/mmol)	168 ± 103	235 ± 189	116 ± 93	0.06
Rejection (any grade; any time)	2 (13%)	0	5 (62%)	0.003
Late (>12 months) rejection	0	0	3 (38%)	0.003
Non-adherence	0	0	2 (25%)	0.01
% GS	42 ± 21	61 ± 12	28 ± 18	0.02
cv-score	1.9 ± 0.7	2.0 ± 1.0	1.13 ± 0.93	0.02
ah-score	2.1 ± 0.83	1.83 ± 0.9	1.38 ± 0.99	NS
i-score	0	0	1.0 ± 0.5	NA*
t-score	0	0	0.8 ± 0.5	NA*

*N/A = Not analysed; i- and t-scores presented in regard to inflammation in non-scarred areas, which was only the case for one of the groups specifically defined a priori. Therefore statistical analysis inappropriate

**Fig 2:**
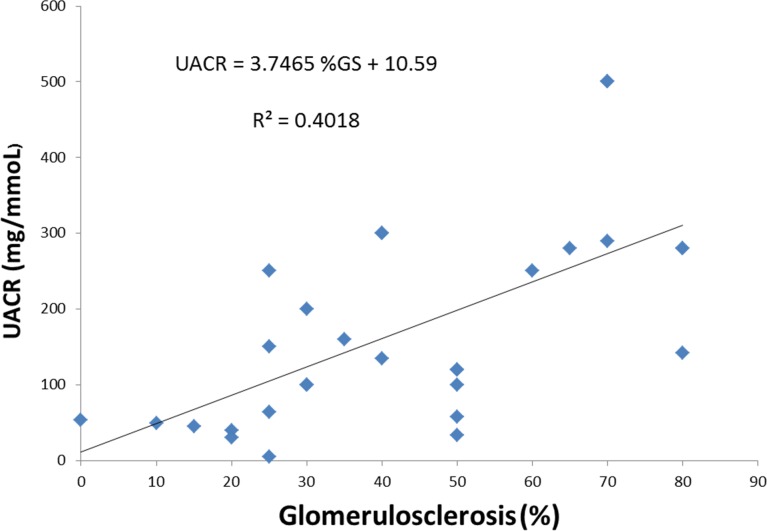
Relationship between proteinuria (early morning “spot” albumin:creatinine ration; UACR) and percentage glomerulosclerosis in patients with IFTA on indication biopsy proximate to graft failure (n = 25).

As defined, these groups displayed notable clinical differences. Specifically, patients displaying IFTA with inflammation in *non-scarred* areas had more likely experienced prior rejection (any grade;any time), late rejection (>12 months post-transplantation), and nonadherence (p≤0.01 for all). No patients in other IFTA groups displayed nonadherence or late rejection. Time to graft failure was shorter, and renal function decline more rapid, in patients with inflammation in non-scarred areas compared with the other groups (p<0.05 for all comparisons). Proteinuria at biopsy was numerically lower when inflammation in non-scarred areas was seen (p = 0.06). Interestingly, many patients with IFTA displayed marked proteinuria, but this was a manifestation of increasing glomerulosclerosis as part of the scarring process; proteinuria was highly correlated with the percentage of globally sclerosed glomeruli on biopsy in these patients with IFTA (r = 0.63; p = 0.01; Fig 2). By definition, this glomerulosclerosis was not a manifestation of recurrent (or *de novo*) glomerular diseases, which were considered as a separate category of graft failures.

Percentage glomerulosclerosis, “cv” and “ah” scores were all lower in biopsies displaying IFTA with inflammation in non-scarred areas compared with the other 2 groups (p = 0.02 for both comparisons). The paucity of patients not treated with CNI precluded evaluation of the role of CNI exposure in the development of IFTA. More patients with graft failure classified as IFTA were treated with ciclosporin than tacrolimus, but this is confounded by the more recent introduction of tacrolimus into clinical practice coupled with the increased time post transplantation at which IFTA graft failures occurred.

#### T-Cell Mediated Rejection (TCMR)

**Fig 3:**
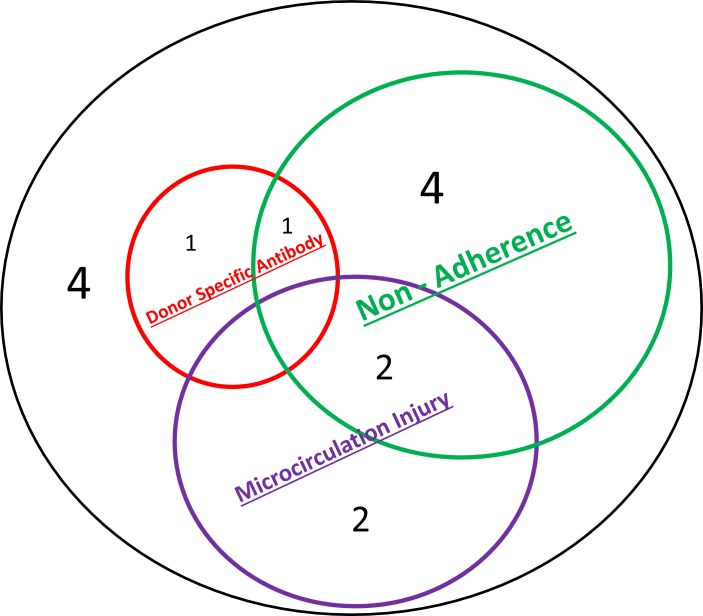
Characteristics of patients displaying TCMR on indication biopsy. Microcirculation injury evident in 4 patients. None displayed circulating donor-specific HLA antibodies or C4d staining and therefore did not fulfil current criteria for ABMR. In addition to interstitial infiltrates these patients displayed the following: glomerulitis n = 1; chronic transplant glomerulopathy n = 1; chronic transplant glomerulopathy with nonadherence n = 1; glomerulitis and chronic transplant glomerulopathy with nonadherence n = 1.

TCMR was seen on indication biopsy in 14 patients. All cases met criteria for Banff 1B rejection. Histological features are shown in Fig 3. Nonadherence was an important factor in TCMR (n = 7). Two features suspicious for ABMR were seen on 6 biopsies, specifically the presence of microcirculatory inflammation (n = 4) or circulating (*de novo*) DSA (n = 2). However, these characteristics were mutually exclusive (Fig 3) and so no biopsy met current criteria for ABMR (note *non*-HLA antibodies were not tested as potential causes of microcirculation inflammation). The other 8 patients displayed TCMR without either DSA or microcirculation injury.

#### Characteristics of patients not undergoing biopsies

Twenty-one grafts failed without an obvious identifiable cause or proximate biopsy. These grafts failed late after transplantation (all beyond 5 years and most beyond 10 years; Fig 1), and represented 21.9% of failures beyond 5 years (21/96). DSA (all *de novo*) was detectable in 5 patients, with 16 demonstrating no DSA. These 2 groups did not differ numerically or statistically for any evaluated demographic or clinical factors, with slow progression to failure and moderate proteinuria at the time of failure (Table 4).

**Table 4:** Characteristics of patients not undergoing transplant biopsy proximate to graft failure.

Characteristic	No Biopsy; DSA negative (n = 16)	No Biopsy; DSA positive (n = 5)	p value
Time to Graft Failure (days)	5518 ± 2147	6582 ± 2147	NS
Age at transplant (years)	36 ± 12	30 ± 16	NS
Age at graft transplant (years)	51 ± 13	49 ± 16	NS
Donor Age	43 ± 13	44 ± 13	NS
Live Donor	2 (13%)	0	NS
eGFR 6 months prior to graft failure (ml/min)	15.0 ± 4.2	12.0 ± 1.8	NS
UACR at graft failure (mg/mmol)	127 ± 64	126 ± 69	NS
Previous rejection	2 (12%)	0	NS
Non-adherence	0	0	NS
Class I DSA	0	1 (20%)	NA
Class II DSA	0	2 (40%)	NA
Class I + II DSA	0	2 (40%)	NA
C1q-binding DSA	0	0	NA

#### Causes of Late, Unselected Graft Failures

**Fig 4:**
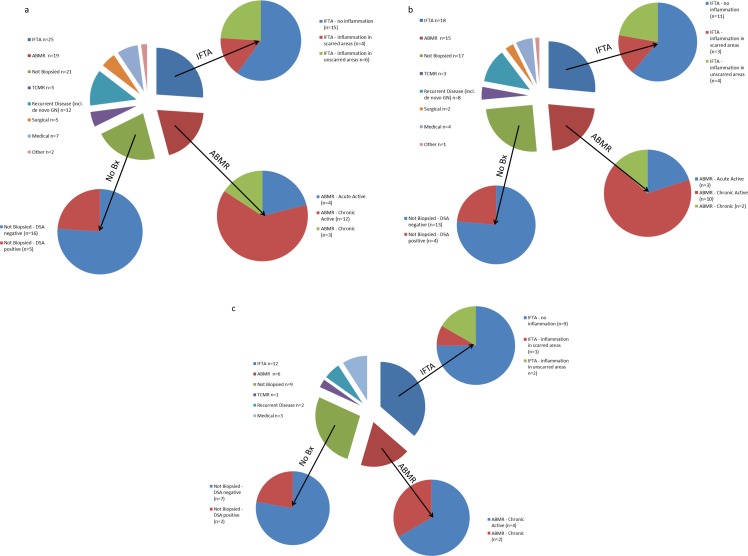
**Breakdown of causes of graft failure beyond 5 years (4a), 10 years (4b) and 15 years (4c) post-transplantation, with each figure representing the graft failure distribution between the time point mentioned and the time of graft failure.** Note therefore that graft failures comprising each summary figure will overlap, but the temporal relationships can be discerned by presenting the data in this way.

The causes of graft failure beyond 5, 10 and 15 years (with absolute numbers) are shown in (Fig 4A–4C). Note that graft failures comprising each summary figure will overlap, but the temporal relationships can be discerned by presenting the data in this way. Although a spectrum of aetiologies is seen, the dominant drivers to late graft failure are ABMR, IFTA and ‘chronic graft attrition’ not undergoing biopsy. Particularly at later times, ABMR is dominated by biopsies demonstrating chronic lesions, and IFTA cases are dominated by those not displaying concomitant inflammation. Beyond 15 years, IFTA was the commonest biopsy finding (n = 12), followed by definite/probable ABMR (biopsy-proven:n = 6; non-biopsied with DSA: n = 2), followed by non-biopsied cases with no detectable DSA (n = 7). With the exception of patient age (whereby patients with graft failures categorised as ABMR or TCMR were younger at the time of transplantation and at the time of graft failure than those classified as IFTA or ‘chronic graft attrition’ [p<0.05 for all comparisons]), no other baseline demographics were associated with differing causes of graft failure.

## Discussion

Very appropriately ABMR is considered a major cause of late graft failure. This study confirms its importance, and refines clinic-pathological correlations in the context of contemporary histological classification and antibody evaluation. Yet the attention to ABMR has been mirrored by the concept that other causes of graft failure, in particular progressive scarring (“IFTA”), are unimportant. The current study opposes this prevailing view by examining unselected patients with true late graft failure, which by virtue of design and duration of previous studies have been incompletely represented hitherto. Acknowledging this heterogeneity informs practice, and suggests individualisation of therapy directed towards the prevailing mechanism.

The close relationship between DSA and histological ABMR speaks to the robustness of the current histological classification [10], including more recently described lesions of ABMR (vasculitis; chronic intimal thickening; C4d-negative ABMR) which were all seen in this cohort. Differences between ABMR subgroups were seen, most notably faster progression to graft failure and a particularly high incidence of nonadherence (66%) in Acute Active ABMR. Interestingly, ah-scores were lower in Acute Active ABMR and in nonadherent patients. Although nonspecific, arteriolar hyalinosis nevertheless represents a component of calcineurin-inhibitor nephrotoxicity, and therefore (inappropriately) lower CNI exposure may underlie this observation, and in part explain previous observations linking arteriolar hyalinosis to *improved* graft outcome [3,7].

C1q-binding DSA were found in 33% of patients with ABMR. Although their frequency was similar across subgroups of ABMR, they were associated with shorter time to graft failure, perhaps representing a marker of more aggressive alloimmunity. This certainly resonates with the brisk rates of failure seen in patients displaying these antibodies previously [13].

Nonadherence was also found in 50% of patients with TCMR-associated graft failure, which was seen at all times beyond 1 month. The paucity of TCMR-related failure beyond 10 years (n = 3) is broadly in keeping with, although not perfectly aligned to, recent findings suggesting complete absence of TCMR on indication biopsies beyond 10 years [14]. Indeed the true contribution of TCMR may be understated given the lack of protocol biopsies and the possible contribution of TCMR in some cases labelled as predominant ABMR. Some patients with TCMR displayed microvascular inflammation, which is a recognised phenomenon. Although (by definition) no circulating anti-HLA DSA was detected, and although it has been said that detection of anti-HLA DSA is a prerequisite for ABMR diagnosis [11], it is conceivable these cases represent ABMR secondary to non-HLA antibodies, which were not tested routinely. But in fact, at least in this series, such misclassification would only pertain to 2 patients (Fig 3). Also not undertaken were gene expression studies to identify “antibody/endothelial interaction”. But again such information would not have improved classification, as cases with DSA displayed no evidence of the (vascular) “tissue” injury required for diagnosing ABMR (Fig 3)[10]. That is not, however, to dispute that gene expression research does continues to refine our understanding of transplantation injury, although its clinical and diagnostic utility is still evolving.

Advanced fibrosis (“IFTA”) in the absence of defined immune/inflammatory lesions was a major cause of graft failure in this series, particularly for late graft failures. Other studies suggest this lesion to be unimportant, although their design was such that IFTA was likely under-represented as discussed above [3–6]. Even so, and in support of our current findings, it should be noted that broadly similar timepoints post-transplantation the prevalence of graft failure from IFTA in the important study from the Mayo Clinic [6] was actually similar to the current study. Specifically, failures due to IFTA *in the absence of identifiable underlying cause* was 5.8% after mean follow-up 5 years versus 5.5% [4/73] within 5 years in the current study. Beyond 5 and 10 years post-transplantation failures became increasingly biased towards IFTA which therefore represented a dominant finding in “true” late graft failure. And it is precisely these failures at these late times which have proven so resistant to the advances and evolution of transplantation practice during the last 25 years [1,2].

Inflammation was seen in some biopsies with IFTA, in the absence of other inflammatory diagnoses. This was either restricted to areas of scarring, or present in non-scarred areas but by definition not meeting criteria for rejection. Subgroup analysis revealed interesting clinico-pathological correlations. Specifically, nonadherence and late rejection episodes were unique to the group with inflammation in *non-scarred* areas, with these patients experiencing brisker functional decline. We contend that this biopsy finding may represent a low-grade alloimmune response, and attention to adherence and immunosuppression is mandatory.

Yet the majority of patients with IFTA displayed either no inflammation or inflammation restricted to scarred areas. The clinical characteristics of patients with these findings were similar, without an abundance of rejection or nonadherence, and similar rate of graft decline. We therefore contend that inflammation within areas of transplant fibrosis may represent a nonspecific feature of the scarring process itself, which indeed is recognised elsewhere [15]. Other studies have demonstrated inflamed scarring within transplants as a component of other progressive inflammatory diseases [16], but this was not relevant in the current study which classified such inflammatory diseases entirely separately. For those patients who did not undergo biopsy, by comparing these patients (Table 4) alongside patients with ABMR and IFTA (Tables 2 and 3 respectively), one can speculate that the non-biopsied patients with DSA (n = 5) most closely resembled Chronic Active or Chronic ABMR, and those without DSA (n = 16) resembled those with IFTA either without inflammation or with inflammation in non-scarred areas.

An important question stemming from the current study is why IFTA may progress in the absence of inflammatory disease, a process previously coined “mysterious dysregulated fibrosis” [4,17]. Recent reports offer new insights into this process and include epigenetic mechanisms resulting in constitutive fibroblast activation [18] and re-expression of embryonic genes from injured tissue resulting in maladaptive downstream fibrogenesis [19]; fibrosis results in local hypoxia, resulting in hypoxic damage to neighbouring tissue and a self-perpetuating cycle of damage [20]; reduced nephron mass with associated glomerular hyperfiltration and proteinuria are mechanisms more specific to the anatomical structure of the kidney [21]. Thus “fibrosis begets fibrosis”, and therapies directed towards this phenomenon, which are emerging in native kidney disease will hopefully prove their worth in transplantation [22]. Finally, the prevalence of severe arteriolar hyalinosis in grafts with IFTA (in particular in when IFTA was present without concomitant inflammation) also speaks to a role for chronic CNI toxicity that should not be forgotten [23–26]. We intentionally avoided attempts to link therapeutic changes with outcome, as conclusions are unlikely to offer insight beyond existing evidence, and may indeed be misleading. We believe it is unlikely that we missed antecedent inflammation (especially ABMR) on biopsies ultimately classified as “IFTA” in light of our biopsy practice, and also the lack of either acute or chronic ABMR (and absent DSA) on such biopsies classified thus.

The results of the current study require interpreting in the context of the changing face of transplant immunosuppression and donor/recipient demographics. The design of the study does not lend itself to interpretation of the influence of changes in clinical practice over time, and the results require careful interpretation in that context. Yet in parallel, a major strength is the examination of “true” late graft failure. It affirms the importance of ABMR in late graft loss, and highlights the importance of progressive fibrosis as important pathway to graft failure and target for intervention. This understanding of the true spectrum of graft failure is an important step in improving clinical practice by individualising patient care.
